# The Liver That Cured Christmas: Case Report of Orthotopic Liver Transplant in a Patient with Hemophilia B

**DOI:** 10.1155/2020/7873803

**Published:** 2020-03-18

**Authors:** Jesse E. Harris, Jonathan L. Balk, Constance M. Mobley, Kirk Heyne

**Affiliations:** ^1^Department of Pharmacy, Houston Methodist Hospital, USA; ^2^Department of Transplant Surgery, Houston Methodist Hospital, USA; ^3^Department of Oncology, Houston Methodist Hospital, USA

## Abstract

Herein, we discuss a case of a 39-year-old male with hemophilia B, who developed end-stage liver disease secondary to nonalcoholic steatohepatitis, that underwent orthotopic liver transplantation (OLT) as a curative means for his liver disease and coagulation disorder. Existing case reports have demonstrated favorable outcomes in patients outside of the United States who received continuous infusions of recombinant factor IX replacement in the perioperative setting after liver transplant. Given limitations in the stability of the recombinant factor IX products in the United States, a dosing strategy was comprised of once daily bolus dosing to achieve satisfactory factor IX levels. Within 48 hours of initial surgery, the patient had sustained factor IX levels above 70% of normal and the patient required no further dosing of factor IX products to date. This strategy helped facilitate less frequent dosing as well as achieved targeted factor levels while synthetic function of the transplanted liver recovered.

## 1. Introduction

Abnormal bleeding disorders have been described in young male patients who have died due to excessive blood loss from relatively minor injuries since the 10^th^ century [1]. As early as 1803, it was hypothesized that bleeding disorders may have a component of genetic predisposition [1]. The term “haemorrhaphilia,” later shorted to hemophilia, was introduced in 1828 to describe these coagulation disorders when little was known how to care for these patients [1]. It was not until the 1950s that factor VIII deficiency (hemophilia A) and factor IX (hemophilia B) were delineated [2]. Factor IX deficiency, also known as “Christmas disease” after being identified in a male child surnamed Christmas [2], occurs in approximately 1 in 30,000 males worldwide [3]. This rare form of hemophilia results from a sex-linked, recessive trait caused by mutations in the factor IX gene of the X chromosome [3].

Historically, management of bleeding episodes in patients with hemophilia relied upon donor plasma-derived factor concentrates and whole blood transfusions. Prior to 1985, viral inactivation processes were not in place for donated blood and plasma. Patients who received frequent transfusions, such as hemophiliacs, were at an increased risk of contracting viral hepatitis which could lead to cirrhosis [4, 5] and ultimately the need for orthotopic liver transplantation (OLT). Liver transplantation as a curative means for cirrhotic patients with hemophilia was first described in 1985 [6].

Several case reports have discussed curative treatment of hemophilia B with OLT in patients who contracted hepatitis C acquired from blood transfusions [7–10]. These reports describe recombinant factor IX therapy using bolus dosing followed by continuous infusions of as a bridge until the newly transplanted liver begins to produce and maintain adequate factor IX levels [11, 12].

Currently, limited literature exists that discusses the use of recombinant factor IX in patients with hemophilia B who undergo OLT for nonviral etiologies of liver cirrhosis. Additionally, limited guidance is available for bolus-based regimens with currently available recombinant factor IX products available in the United States. Herein, we describe the case of a 39-year-old male patient with hemophilia B, nonalcoholic steatohepatitis (NASH), and hepatocellular carcinoma (HCC) who underwent OLT and successfully bridged to sustained normal factor IX levels using intermittent bolus dosing of recombinant factor IX.

## 2. Case Summary

JJ was a 39-year-old male with hemophilia B who was initially diagnosed with cirrhosis secondary to NASH at age 33. His baseline factor IX levels prior to transplantation were 10–22% of normal. The patient had a history of exposure to recombinant factor IX prior to various procedures such as dental procedures, lung and liver biopsies, and transcatheter chemoembolization of the hepatic artery. At age 39, the patient developed HCC and was admitted to Houston Methodist Hospital for liver transplant. On admission, the patient's Model for End-stage Liver Disease (MELD) score was 30 with HCC exception points. One month prior to admission, his factor IX activity level was 12% of normal.

Based on the most recent factor IX levels, a plan was formulated to administer an 80 unit/kg bolus followed by 60 units/kg continuous infusion with a goal factor IX level of 100–120% of normal over 72 hours. Due to limitations in stability of the recombinant factor IX available in the United States, a continuous infusion was not feasible given manufacturer recommendations for stability of 3 hours after initial reconstitution. After successful OLT, the dosing strategy was modified after a discussion with the hematologist, surgical team, anesthesia, and clinical pharmacists. After initial 80 units/kg bolus was to be given perioperatively, the factor IX dosing would be reduced to 20 units/kg per day postoperatively and thromboelastography would be utilized to guide blood product transfusions moving forward. Additional bolus dosing of recombinant factor IX would be permissible in the event of continued bleeding postoperatively.

The patient underwent OLT and required a significant amount of blood products in addition to factor concentrates to obtain hemostasis given the underlying level of coagulopathy. It was estimated that the patient experienced 13 liters of blood loss intraoperatively and received 20 units of packed red blood cells, 20 units of fresh frozen plasma, 20 units of cryoprecipitate, 2,132 units of recombinant fibrinogen concentrate, and a bolus of 80 units/kg of recombinant factor IX intraoperatively in total. Given the level of coagulopathy and volume of blood products needed to achieve hemostasis, temporary closure of the abdomen was performed after hepatic anastomosis with a plan to go back to the operating room when more stable. The patient was packed with laparotomy pads and transferred to the intensive care unit where he remained in stable, but critical condition.

**Figure 1:**
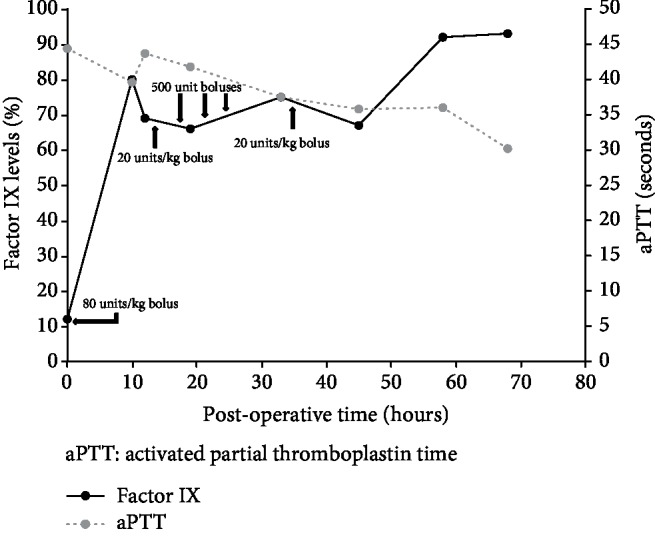
Detailed course of factor IX and serum aPTT levels.

After the first stage of OLT and initial 80 units/kg bolus of recombinant factor IX, the patient's factor IX level increased to 80% of normal on post-op day (POD) 0. The patient was taken back to the OR for exploratory laparotomy 8 hours later due to continued blood loss. Intraoperatively, the patient required 6 units of fresh frozen plasma and 9 units of packed red blood cells during hematoma evacuation and repacking of laparotomy pads. His factor IX replacement was reduced to 20 units/kg daily to maintain target levels. Prior to the patient undergoing the second stage of OLT for biliary anastomosis on POD 1, the patient's factor IX levels had decreased to 66% of normal and was given 3 separate 500-unit boluses over a 12-hour period. After the second stage of the liver transplant for biliary anastomosis on POD 1 was complete, the patient was given a 20-unit/kg dose of factor IX. On POD 2, the factor IX level was 92% of normal indicating resolution of hemophilia and normal factor IX synthesis in the newly transplanted liver. No further dosing of exogenous factor IX was required. A full report of the patient's factor IX levels, dosing strategy, and PTT can be seen in Figure 1. The patient had no further bleeding complications and was transferred to a step down unit on POD 7 with discharge to home on POD 12.

## 3. Discussion

Several case reports, as well as retrospective studies, have shown resolution of underlying deficiencies of factor VIII and or IX synthesis in hemophiliac patients who undergo OLT for correction of underlying liver disorders [13–16]. Although there are guidelines present for perioperative management of exogenous factor replacement in hemophiliac patients undergoing procedural surgical intervention [17], limited guidance is available for major surgery, specifically liver transplantation.

With this case, we describe a successful orthotopic liver transplant in a patient with hemophilia B utilizing intermittent bolus infusions of recombinant factor IX as a bridge to adequate factor IX level synthesis of the transplanted liver. Kinetic calculations were utilized to estimate desired factor IX levels and then adjusted based on a patient-specific response, as recommended by Shapiro et al. [18], to balance the response estimated from the recombinant factor IX administrations with the synthetic function of the transplanted liver. While continuous infusions would have provided a more predictable kinetic profile, this approach was not ideal for stability and nursing administration purposes. Given an 8–12-hour activity half-life of the factor IX product available at our institution, a greater degree flexibility was available using an intermittent bolus regimen.

The recombinant factor IX product used exclusively at our institution is BeneFIX® (Wyeth Pharmaceuticals Inc.) which carries a recommended stability of 3 hours post initial reconstitution [19]. Given the short stability, our team opted to once daily bolus dose infusions in lieu of continuous infusions to achieve target factor IX levels postoperatively. Considerations were made to split the total daily-calculated doses to be given throughout the day to approximate continuous infusion strategies. However, this approach could potentially cause delays in therapy and interfere with the need for blood product transfusions that were ongoing. Ultimately, the strategy to use once daily bolus infusions allowed for shortened infusion times and achieved targeted factor IX levels within 48 hours of OLT. The patient experienced no further complications related to the synthetic function of the transplanted liver and is doing well to this date.

In conclusion, our report demonstrates a potential dosing strategy that resulted in satisfactory factor IX levels post OLT in a patient with hemophilia B and end-stage liver disease. Our approach to utilize preoperative bolus dosing followed by postoperative once daily infusions to obtain factor IX levels above 90% of normal as a bridge until synthetic function of the newly transplanted liver could result in sustained levels and ultimate cure of his hemophilia B.
